# 

**Published:** 1902-08-16

**Authors:** 


### NOTICE TO CORRESPONDENTS.

All MSS., Letters, Books for Review, and other matters intended for the Editor should be addressed The Editor, "The Hospital," The Hospital Building, 28 & 29 Southampton Street, Strand, W.C.

The Editor cannot undertake to return rejected MS., even when accompanied by stamped directed envelope.

**Notice to Advertisers and Subscribers.**

All Advertisements, Orders for Copies of The Hospital, and Business Communications should be addressed to **THE MANAGER, (Not to the Editor)**, The Hospital, 28 & 29 Southampton Street, Strand, London, W.C.

The Cost of Subscription, post free, is as follows.—Per week, 2½d.; per quarter, 2s. 8d.; per half-year, 5s. 3d.; per annum, 10s. 6d. Foreign:—Per annum, 15s. 2d., payable, in all cases, in advance.

**NOTICE.—Vol. XXXI. of "The Hospital" (October 1901, to March 1902), in handsome cloth binding is now on sale at the office of this Journal, price, 7s. 6d. Cases for binding the Numbers contained in this Volume 1s. 6d. Index to Vol. XXXI., 2½d., post free.**

**The Monthly Part for August is now ready. Price 6d., by post 8d.**

### The Student of Medicine.

### APPOINTMENTS.

Arthur Burton, M.D., appointed Certifying Factory Surgeon for the Cromer District. Frank Dendle, M.B., C.M., D.P.H.Edin., appointed Divisional Surgeon to Isleworth Section of T. Division Metropolitan Police, and Medical Officer to Spring Grove College. John D'Ewart, M.R.C.S., L.R.C.P., appointed House Physician of the Manchester Royal Infirmary. S. H. Johnson, M.R.C.S., L.R.C.P.Lond., appointed District Medical Officer of the Sculcoats Union. R. E. Moorhead, L.R.C.P., L.R.C.S.Edin., appointed District Medical Officer of the Bath Union. W. A. Valentine, M.D., B.Ch.Univ.Dubl.Trin.Coll., appointed Surgeon and Agent, Sick Quarters, under the Admiralty at Appledore, Devon. Tom. Walker, M.R.C.S., L.R.C.P., appointed House Surgeon of the Manchester Royal Infirmary.

### Standard Books of Reference.

"The Nursing Profession: How and Where to Train." 2s. net; post free 2s. 4d.
"Burdett's Official Nursing Directory." 3s. net; post free, 3s. 4d.
"Burdett's Hospitals and Charities." 5s.
"The Nurses' Dictionary of Medical Terms." 2s.
"Burdett's Series of Nursing Text-Books." 1s. each.
"A Handbook for Nurses." (Illustrated). 5s.
"Nursing: Its Theory and Practice." New Edition. 3s. 6d.
"Helps in Sickness and to Health." Fifteenth Thousand. 5s.
"The Physiological Feeding of Infants." 1s.
"The Physiological Nursery Chart." 1s.; post free, 1s. 3d.
"Hospital Expenditure: The Commissariat." 2s. 6d.

All these are published by the Scientific Press, Ltd., and may be obtained through any bookseller or direct from the publishers, 28 and 29 Southampton Street, London, W.C

